# Cold damage from wax deposition in a shallow, low-temperature, and high-wax reservoir in Changchunling Oilfield

**DOI:** 10.1038/s41598-020-71065-z

**Published:** 2020-08-26

**Authors:** Jun Xie, Xiao Hu, Hui-zhen Liang, Meng-qi Wang, Fa-jun Guo, Shu-juan Zhang, Wu-chao Cai, Rui Wang

**Affiliations:** 1 College of Earth Science and Engineering, Shandong University of Science and Technology, Qingdao, 266590 Shandong China; 2 College of Mechanical and Electronic Engineering, Shandong University of Science and Technology, Qingdao, 266590 Shandong China; 3 School of Environment and Planning, Liaocheng University, Liaocheng, 252000 Shandong China; 4 Research Institute of Exploration and Development of Huabei Oilfield Company, CNPC, Renqiu, 062552 Hebei China

**Keywords:** Geochemistry, Energy science and technology

## Abstract

Wax deposition is an important factor that influences oil production for high-wax crude oilfield. There are few studies on the formation damage by wax deposition, especially cold damage to the shallow low-temperature reservoir. With laboratory tests conducted on reservoir oil and cores of Changchunling Oilfield, this study aims to experimentally investigate the influence of temperature variations on characteristics of oil–water percolation and cold damage mechanisms, as well as the relative permeability of high-wax reservoirs. Experimental results show that seepage flow of high-wax crude is significantly sensitive to temperature-wax deposition evidently increases, whereas the cold damage such as the pore-throat radius and relative permeability sharply decrease with the decline in formation temperature. The research results can be applied to enhance oil recovery of high-viscosity or high-wax oilfields.

## Introduction

With the reduction in available conventional oil, heavy oil is becoming an important resource. The majority of crude oil fluids contain a certain proportion of heavy hydrocarbon compounds^1^. The deposition of heavy organic compounds from crude oil can be attributed to thermodynamic equilibrium between the porous medium and the fluid breakdown during oil mining^2,3^. The solid-phase material may induce reservoir blockage, resulting in low recovery and seriously affecting oil or gas development^4^. Changchunling Oilfield is a recent discovery in Jilin petroliferous area, whose burial depth ranges from 210 to 350 m. The general formation temperature is 17.6 °C, and the original formation pressure is 1.89 MPa. The highest wax content of crude oil is 31.6%, and viscosity (50 °C) varies from 21.6 to 75.6 mPa s. Due to serious wax deposition within reservoirs, the current exploitation of this oilfield is mainly characterized with low single-well production capacity, low formation pressure, and poor recovery^5^. To identify effective mining methods, thermal recovery experiments have been conducted in this special field since 2007, including a steam drive, hot water flooding and fire flooding. However, no satisfactory results have been achieved.

**Figure 1:**
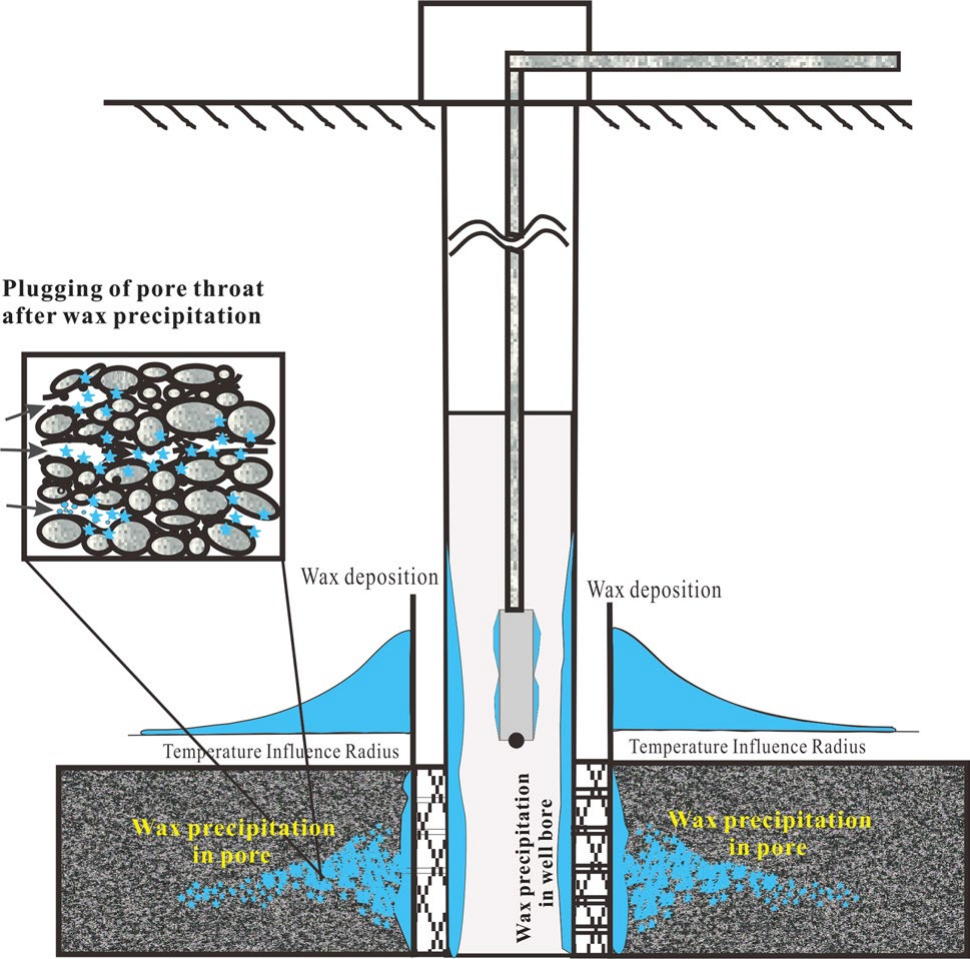
The diagram of formation damage caused by wax deposition.

Cold damage from wax deposition mainly occurs in the reservoir, well bores, pipelines, and other production equipment (Fig. 1). Wax deposition in the reservoir is an important factor that influences well productivity for high-wax crude oilfield^6,7^. Since there are few studies on reservoir damage causing by wax deposition, the degree and scope of reservoir damage remain unknown, and no available experimental methods and effective protection measures have been put forward. Therefore, studies on wax deposition in porous media are of theoretical and practical significance, especially in the improvement of oilfield recovery.

## Experimental procedures

**Figure 2:**
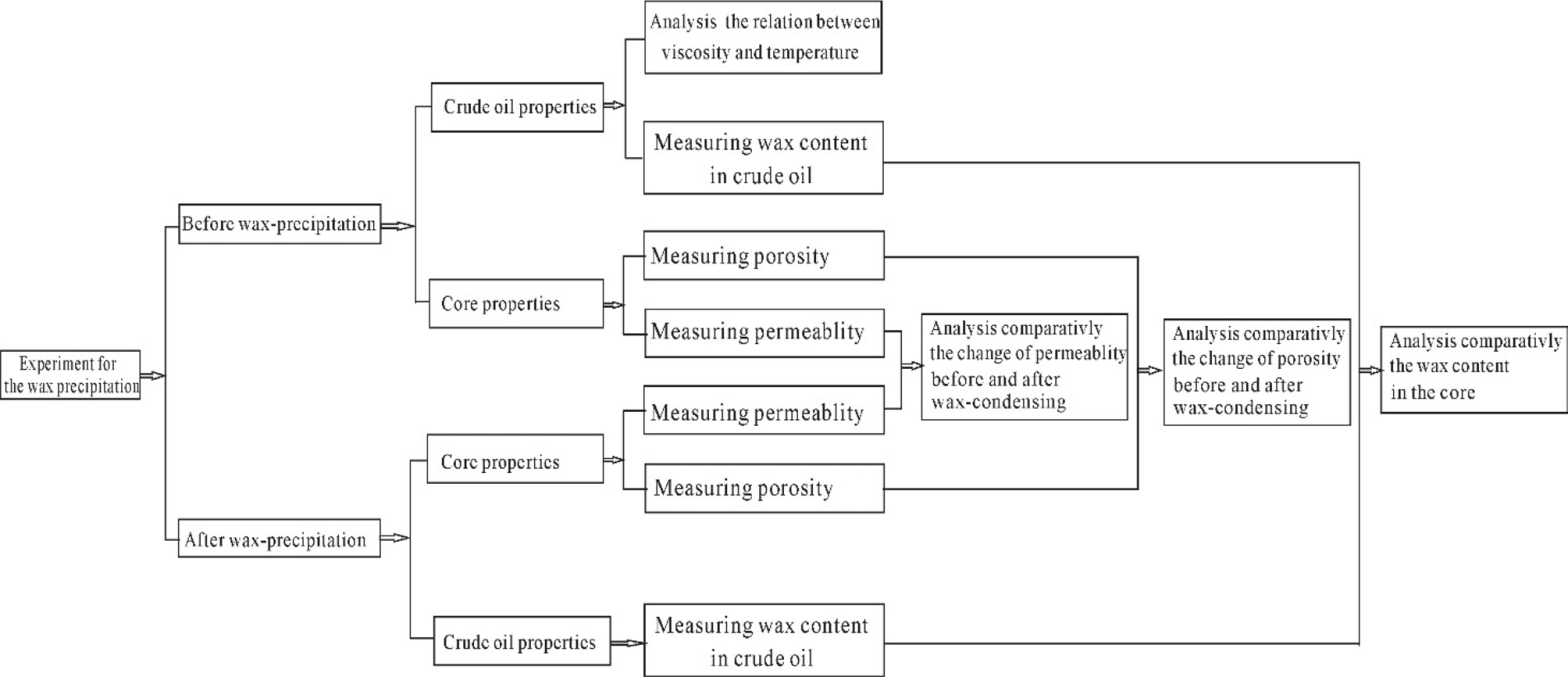
Experimental flow chart for the wax deposition.

The extent of damage to the reservoir wax deposition mainly depends on characteristics of crude oil and reservoir. Samples of crude oil and core are taken from corresponding research area in Changchunling Oilfield. Figure 2 is an experimental flow charts for the wax deposition, which focuses on crude oil and core properties before and after wax deposition. The contrastive analysis in experimental results, such as wax content in crude oil, porosity and permeability, demonstrates the extent and influence factors of cold damage to shallow oil reservoir in low temperature.

## Properties of crude oil in the experiment

### Rheological properties of crude oil

The rheological properties of waxy crude oil are strongly sensitive to temperature, and the freezing point and viscosity are important factors to seepage flow and well productivity. A rotational viscometer is one of the commonly-used instruments to determine the crude oil viscosity at varying temperatures^8,9^. Detailed process of measuring the viscosity of crude oil by the rotational viscometer can refer to the method of SY/T 0520-2008 (China Industry Standard). It should be noted that if the sample shows the characteristics of non-Newtonian fluid, at least 5 shear rates should be tested at intervals of 2 °C for each measuring point.

Rotational viscometer (Rheotest-2) is used to measure the rheological properties of crude oil. But it’s complex to determine the shear rate when measuring non-Newtonian fluids. The rheological equation can be expressed in power law:

1 $$\tau =K{\dot{\gamma }}^{n}$$

where τ is shear stress, Pa, K is viscosity coefficient, n is rheological index.

$\dot{\gamma }$ is shear rate, which can be defined as:

2 $$\dot{\gamma }=\frac{2}{n}\Omega {\left\{\left[{\left(\frac{1}{{R}_{2}}\right)}^\frac{2}{n}-{\left(\frac{1}{{R}_{1}}\right)}^\frac{2}{n}\right]{r}^\frac{2}{n}\right\}}^{-1}$$

where R_1_ is the radius of the outer cylinder, R_2_ is the radius of the inner cylinder, Ω is angular velocity of rotation, r is radius of the cylinder clearance.

When n = 1, Newton fluid can be expressed as^10^:

3 $${\dot{\gamma }}_{1}=2\Omega {\left\{\left[{\left(\frac{1}{{R}_{2}}\right)}^{2}-{\left(\frac{1}{{R}_{1}}\right)}^{2}\right]{r}^{2}\right\}}^{-1}$$

When $\delta =\frac{{R}_{2}}{{R}_{1}}$, r = R_2_, Newton fluid can be simplified as:

4 $$\dot{\gamma }_{1} = \frac{\frac{2}{n}}{{1 - \delta^{\frac{2}{n}} }}\Omega$$

For non-Newton fluid:

5 $$\dot{\gamma } = \frac{2\Omega }{n}\left[ {\overline{x}^{\frac{2}{n}} \left( {1 - \delta^{\frac{2}{n}} } \right)} \right]^{ - 1}$$

Based on the results achieved from experiments and calculations, it can be concluded that the shear rate has nothing to do with both non-Newton and Newton fluids at a certain radius r_m_. Thus, the correction formula of shear rate for non-Newton fluid is:

6 $$\dot{\gamma } = \left( {\frac{1}{{\overline{x} }}} \right)^{2} \dot{\gamma }_{1}$$

The data $\overline{x}$ achieved by three measurement systems of rotational viscometer can be shown in Table 1.

**Table 1:** Correction coefficient of the shear rate for non-Newton fluid.

System	δ	$\overline{x}$	$(1/\overline{x} )^{2}$
S_1_	0.98	1.020	0.960
S_2_	0.94	1.040	0.924
S_3_	0.81	1.100	0.826

The amount of wax in the crude oil will affect the viscosity of the fluid, and then affect the rheological, as showed in Eqs. (1)–(5) in the text, and finally affect the amount of wax precipitation in the reservoir. This paper analyzes the effect of the viscosity of crude oil sample on temperature in the sampling well. The experimental accuracy requirements for the experimental process are detailed as following.

① Using the coaxial cylinder rotary viscometer, the test error of the instrument shall be nothing more than 4%.
② Constant temperature circulator, the temperature fluctuation shall not exceed ± 0.1 °C.
③ Thermometer, mercury thermometer or other temperature measuring equipment with division value not greater than 0.1 °C.
④ Constant temperature bath, electric oven or air flow dryer, sampling vessel, electronic balance sensitivity 0.01.
⑤ *Repeatability test* The same operator shall have the same instrument in the same laboratory and test according to the prescribed method. In continuous time, the difference between the two results of repeated results of the same sample shall not exceed 15% of the average value, so as to ascertain the validity of the test data.

**Figure 3:**
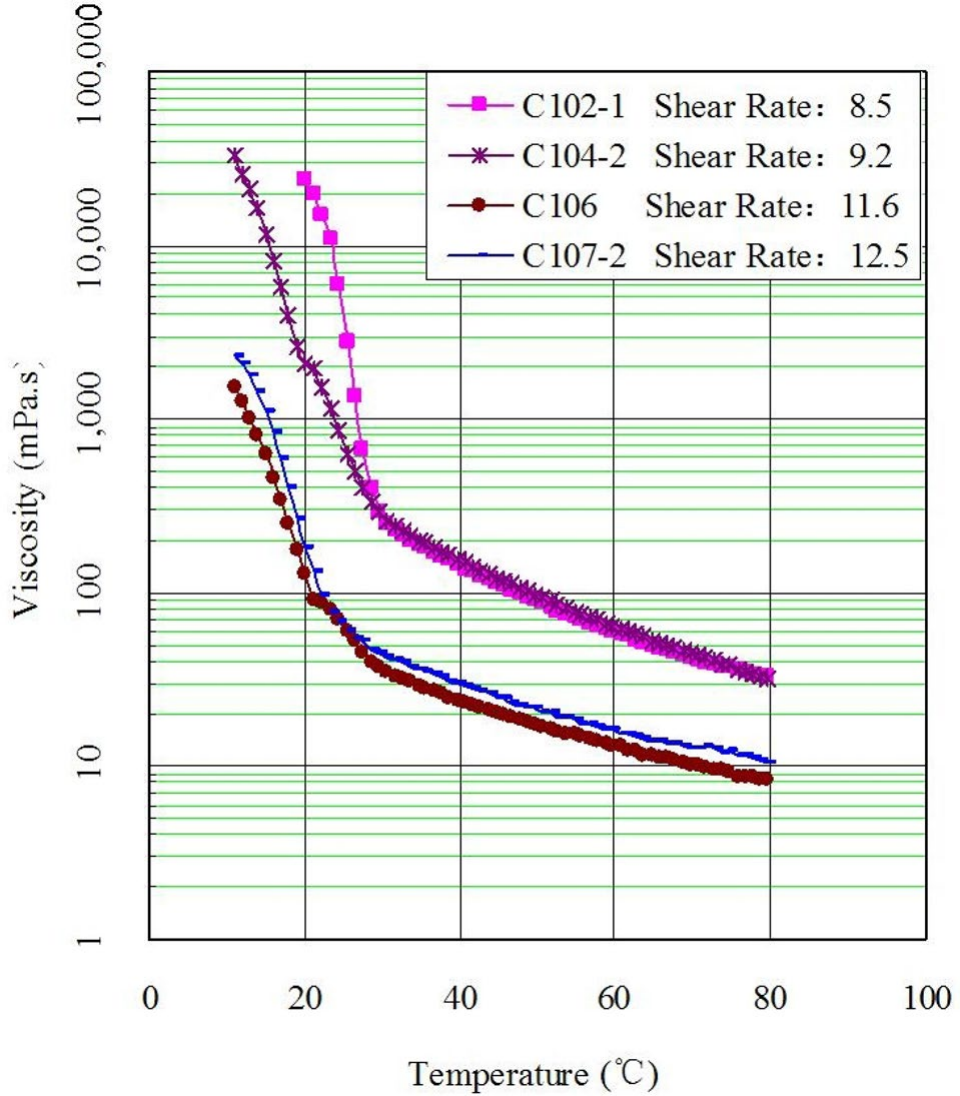
Viscosity–temperature curves of Changchunling oil field.

Viscosity results are shown in Fig. 3. The pour point of crude oil in Changchunling Oilfield is approximately 25 °C.

According to data in Fig. 3, when temperature drops below the cloud point, wax crystals of crude oil increase gradually, and the crude oil begins to show properties of non-Newtonian fluid. When the temperature is close to pour point, both the physical structure and macro property of wax crystals are dramatically changed.

Differential scanning calorimetry (DSC) is currently the best technical approach used in thermal analysis^11^. In this approach, the crude oil sample is heated to a temperature above the wax deposition point, then differential heat flow recorded at each temperature in the cooling process.

**Figure 4:**
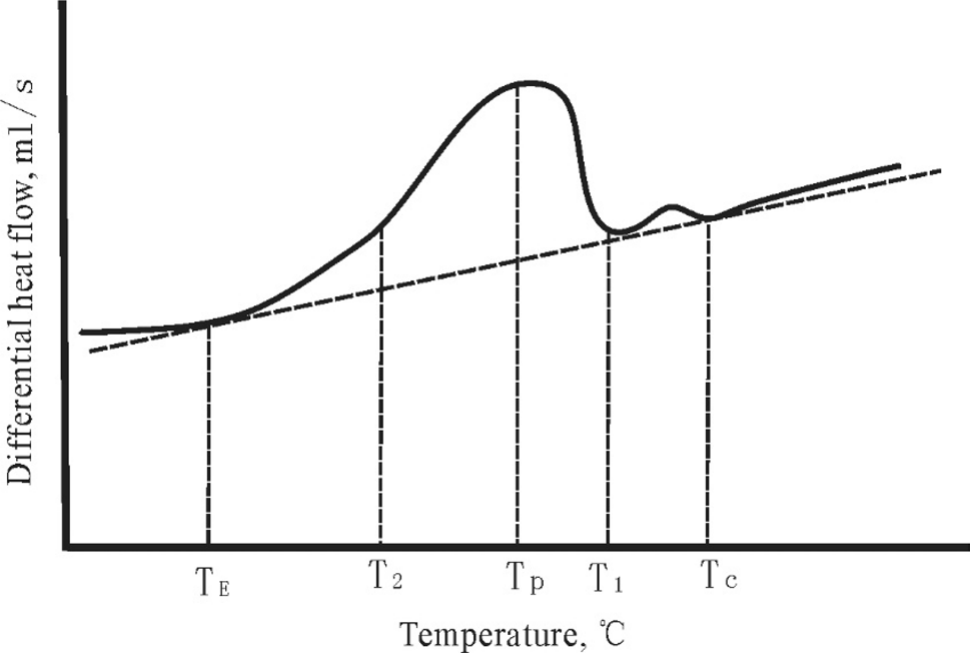
DSC curve of wax deposition.

Experimental analysis indicates that wax deposition of crude oil follows the common law: thermal effects gradually change at varying temperature. The following curve, i.e. Fig. 4, can be used to quantitatively analyze the characteristics of wax deposition, where T_C_ and T_E_ refer to starting temperatures and ending temperature, respectively, T_P_ peak temperature, T_1_–T_2_ peak interval and enthalpy of wax deposition.

Table 2 is the temperature data of wax deposition and dissolve wax of waxy crude oil from Changchunling Oilfield. The wax deposition point is lower than the dissolve point by approximately 10–20 °C, which indicates that oil exploitation becomes more difficult after wax deposition in the reservoir. Thus, a higher temperature than wax deposition point is required to enhance oil recovery.

**Table 2:** The test temperature of wax deposition and dissolve of waxy crude.

Number	Well	Initial temperature of wax deposition(°C)	Peak temperature of wax deposition (°C)	Initial temperature of wax melting (°C)	Peak temperature of wax melting (°C)
1	C112	25.8	20.7	30.2	34.5
2	C110	25.9	15.2	32.8	37.1
3	C107-3-1	30.1	16.5	34.3	39.6
4	C107-1-6	26.1	17.1	36.4	41.3
5	C106	26.6	14.5	31.7	35.4
6	CP2	22.7	11.3	30.1	35.2
7	C109	28.6	20.5	29.8	32.7
8	C109-7-13	25.3	19.9	30.6	33.3
9	C109-2-8	26.7	21.2	29.2	32.8
10	C109-8-14	24.2	20.8	31.1	33.2

**Figure 5:**
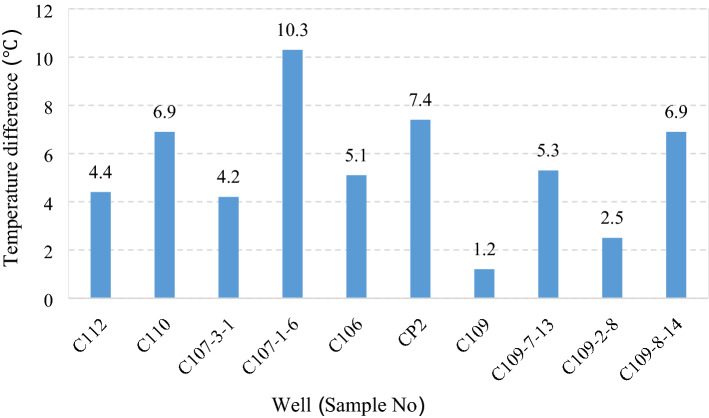
Histogram of wax precipitation temperature and wax dissolution temperature difference of different samples.

Four representative oil wells C110, C109, C107-3-1 and C107-1-6 are selected in the text. It can clearly reflect the influence of temperature on viscosity, wax deposition, porosity and permeability of Changchunling oilfield. The supplementary experimental purpose of Table 2 is to obtain the temperature relationship between wax precipitation and re-dissolution after wax deposition. Wax deposition and re-dissolution after wax deposition are not a simple reverse process. The temperature of wax melting is often higher than that of wax precipitation. The sample data of four wells are relatively small, and the samples of six wells are supplemented for experiments. The temperature difference between wax melting and wax precipitation is mostly between 4 and 10  °C, which can reflect the general law more, and has a wide reference value. Histogram of wax precipitation temperature and wax dissolution temperature difference of different samples as showed in Fig. 5.

### Wax deposition tests

The basic data of the experimental samples in the study area are as following.

The average density of crude oil (50 °C) in block C107 is 0.8905 g/cm^3^, the viscosity of crude oil (50 °C) is generally 21.6–75.6 mPa s^5^, the average viscosity of crude oil is 46.05 mPa s, the average freezing point is 14.6 °C, and the formation temperature in the middle of the oil layer in well C107 is 17 °C.

The average density of crude oil (50 °C) in block C109 is 0. 8575 g/cm^3^, the viscosity of crude oil (50 °C) is generally 19.6–24.1 mPa s, the average viscosity of crude oil is 21.86 mPa s, the average freezing point is 18.2 °C, and the formation temperature in the middle of the oil layer in well C107 is 22 °C.

#### Testing the amount of wax deposition

Theoretically, the amount of wax deposition is related to many factors, such as crude composition, temperature, driving the pressure gradient, oil flow rate, and so on.

Testing process of the amount of wax deposition for core samples at different temperature is as follows: First, test the wax content (ω_1_) of representative crude oil samples in Changchunling Oilfield. Detailed measuring process refers to the method of SY/T 0537-2008 (China Industry Standard). Second, pump the same oil sample used in the first step into the core sample at a high injection pressure. The experiment lasts three days with temperature varying from 50 to 5 °C. Test the wax content (ω_2_) of oil samples. The amount of wax deposition containing in core sample can be achieved by (ω_1_–ω_2_).

**Figure 6:**
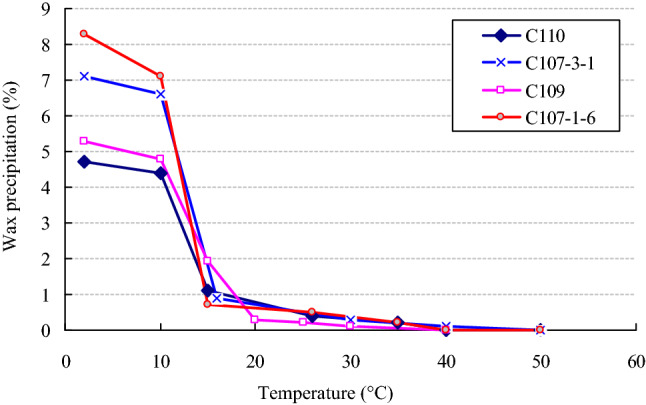
The wax deposition volume–temperature curves.

Figure 6 shows reflect changes of wax deposition for different well oil samples at different temperature. It can be concluded that wax deposition increases evidently as the temperature drops below 15 °C.

#### SEM inspection for the presence of wax

**Figure 7:**
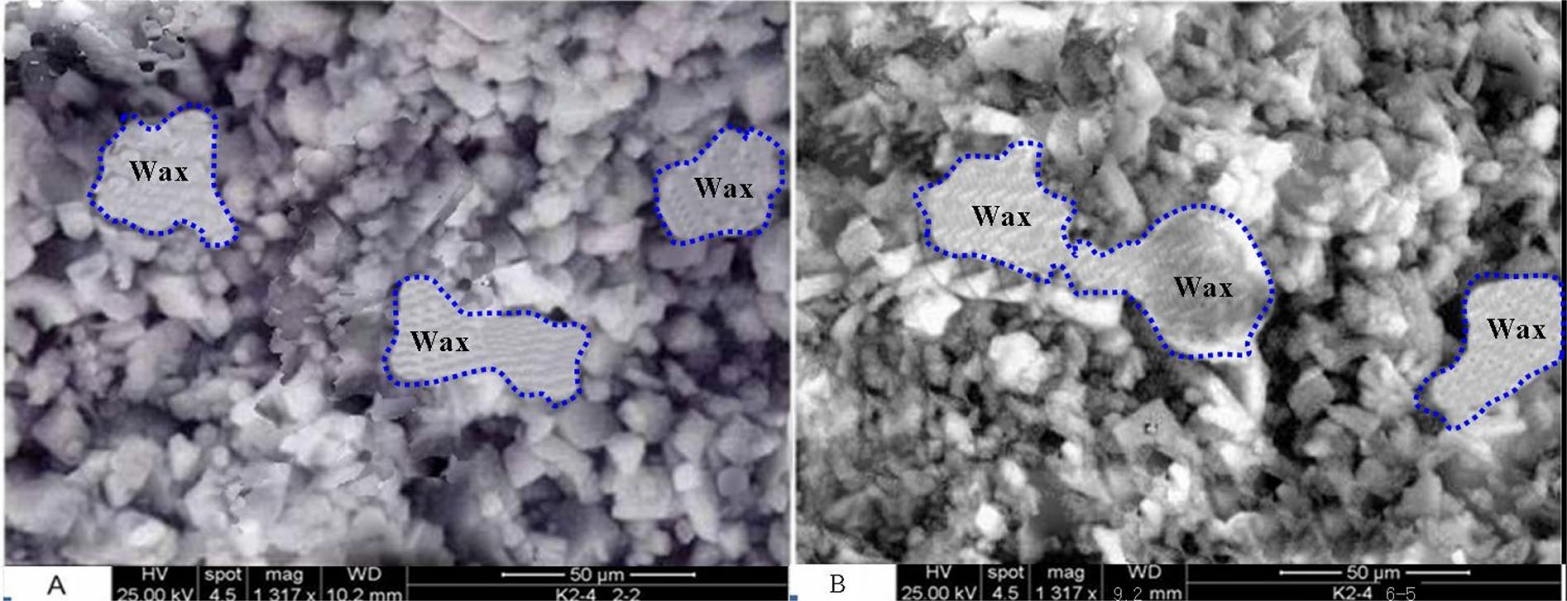
Photos of wax in pore space.

The sample is kept cold as it is removed from the core holder for the Scanning Electron Microscope (SEM) analyses. Chips are broken from the center of the plug for SEM imaging. The core material is maintained at 15 °C during SEM imaging^12^. Presence of wax is confirmed by photomicrographs. Wax distribution is uneven, with many pores apparently free of wax. Wax appears as masses rather than crystals^13^. Some of the wax structures identified are significantly larger than average rock pore dimensions, and appear to adhere to rock mineral surfaces, as showed in Fig. 7.

### Core properties in the experiment

Before oilfield exploitation, under the condition of high temperature and high pressure in the formation, most of the crude oil buried in the formation is liquid, the wax is completely dissolved in the crude oil, and the reservoir fluid and porous medium are a thermodynamic equilibrium state. Once the oilfield is put into development and production, this balance will be undermined, and the reservoir pressure and temperature will gradually decrease. When the pressure and temperature decrease to a certain extent, the wax is separated from the dissolved state of crude oil, forming crystalline particles, and attached to the wellbore and the formation near the wellbore, resulting in the rapid reduction of reservoir porosity and permeability^14^. The Changchunling oilfield is a shallow superior wax bearing oilfield in Jilin Oilfield. The top of the reservoir in the study area is 200–350 m deep, and low temperature and low pressure of the oil layer are caused by shallow burial. The formation temperature in the middle of an oil layer of well C107 is 17 °C, and the formation temperature in the middle of the oil layer of well C109 is 22 °C. Under the condition of oil layer, the mobility of crude oil is poor. In the process of exploitation, the reservoir is the most likely to wax out and produce cold damage, resulting in low or even no conventional production capacity, which seriously affects the normal production of oilfield and restricts the effective utilization of reserves.

#### Porosity changes with temperature during wax deposition

The method of SY/T 5336-2006 (China Industry Standard) should be followed to analyze the porosity changes at different temperature before and after wax deposition. It’s necessary to note that low-temperature dried process is required to prevent the wax from melting in the process of measuring the porosity after wax deposition.

**Figure 8:**
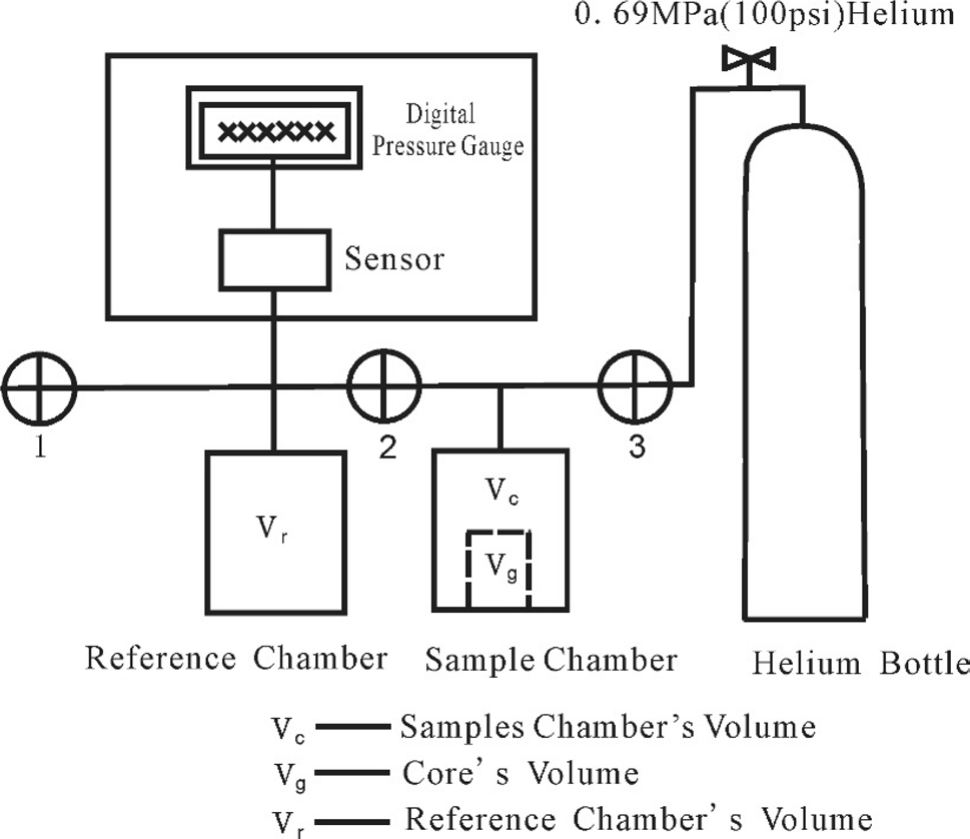
The schematic diagram of porosity measuring device.

Pore becomes smaller after wax deposition. Core porosity is tested under high pressure (> 4 Mpa) and high purity. As is showed in Fig. 8, Helium is injected into the chamber until wax core samples are saturated. After equilibrium pressure is determined, open the valve to make gas spread into the reference chamber. Then calculate the pore volume according to Boyle’s law. The detailed procedure can be described as following:

First measure the atmospheric pressure (P_0_) when valve 1 is open and valve 2 and 3 are closed. Then close valve 1 and put the core sample with wax deposition into the sample room; open the valve 3, high pressure gas of 4–5 Mpa (absolute pressure) filled into samples room, 2–5 min later, close the valve 3, balance 2–3 min later, open the valve 2, measured the pressure P_3_, open the valve 1, gas diffusion from the sample chamber to the reference chamber, equilibrium 3–5 min later, determined P_2_,according to Boyle’s law:

7 $${\text{P}}_{0} {\text{V}}_{{\text{r}}} + {\text{ P}}_{{3}} \left( {{\text{V}}_{{\text{p}}} + {\text{ V}}_{{\text{d}}} } \right) \, = {\text{ P}}_{{2}} \left( {{\text{V}}_{{\text{r}}} + {\text{ V}}_{{\text{p}}} + {\text{ V}}_{{\text{d}}} } \right)$$

where V_P_ is pore volume of wax samples, V_r_ the reference chamber volume, V_d_ dead pipeline volume. V_P_ can be obtained:

8 $${V}_{P}=\frac{({P}_{2}-{P}_{1}){V}_{r}-({P}_{3}-{P}_{2}){V}_{d}}{{P}_{3}-{P}_{2}}$$

Then the rock porosity can be expressed as:

9 $$\Phi =\frac{{V}_{P}}{{V}_{g}}$$

where V_g_ is the core volume.

**Figure 9:**
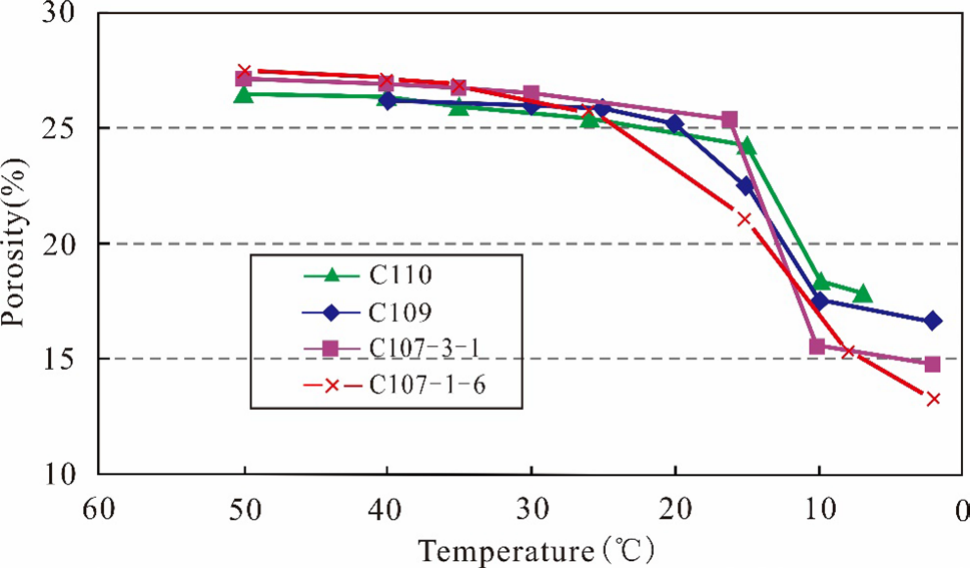
The porosity–temperature curves.

Wax deposition on the surface of porous medium directly results in the reduction of reservoir porosity^15,16^. The influence of wax deposition on porosity is quantitatively analyzed according to experimental results at different temperature. Figure 9 represents the effects of wax deposition on porosity in the three representative wells C109, C110, and C107-3-1. It reveals that reservoir porosity sharply decreases, and wax content dramatically increases with the falling reservoir temperature, especially at 10–20 °C.

#### Permeability changes with temperature during wax deposition

Since non-Newtonian fluid is not a simple single-phase flow, the reservoir in different phases has different internal temperature and pressure^17^. Flow or heat transfer process occurs. In this process, momentum transfer law is complex.

The changing rate of permeability is defined to indicate the influence of temperature to permeability^18^

10 $${R}_{k}=\frac{\Delta K}{K\Delta T}$$

R_k_ is the permeability changing rate, 1/°C; ΔK is the permeability difference, 10^–3^ μm^2^; ΔT is the temperature difference, °C.

Core flooding tests are conducted at 50, 40, 35, 30, 25, 20, 15, 10, 5 °C separately to analyze the waxy crude oil flow in a porous medium at a different temperature. Thus, oil effective permeability can be achieved to represent the flow ability of oil.

**Figure 10:**
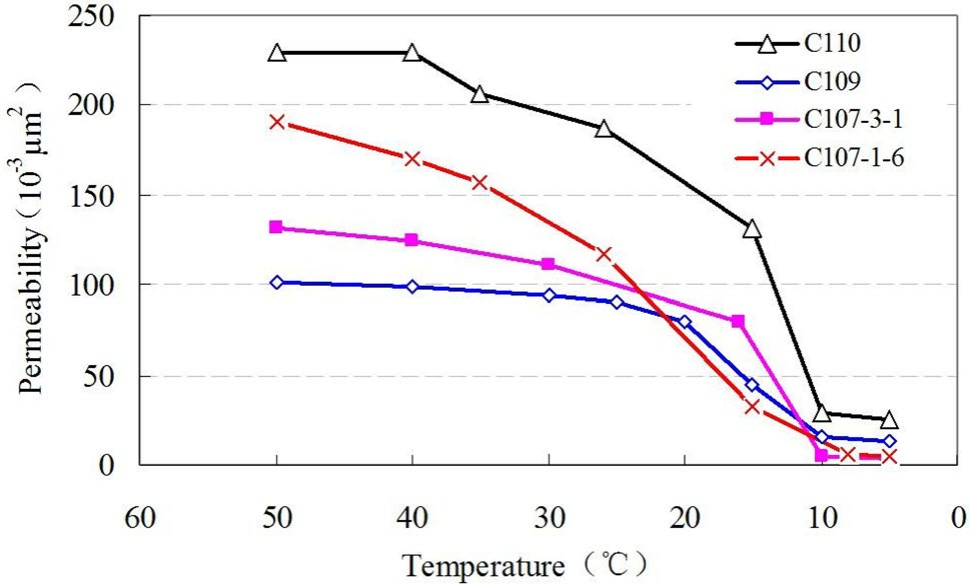
The permeability–temperature curves.

Figure 10 is the permeability change with oil temperature. Oil effective permeability decreases with temperature declines. It shows that for high-permeability cores, the permeability change is small, while for low-permeability cores, the change is much sharper with temperature.

For waxy crude oil seepage flow, effective permeability is a multi-variable function^19^:

11 $${K}_{e}=f\left({K}_{0},T,{V}_{c}\right)$$

K_e_ is the effective permeability, 10^–3^ μm^2^; K_0_ is the original permeability, 10^–3^ μm^2^; T is the temperature, °C; V_c_ is the cumulative oil flow, PV.

## Results and discussion

### Seepage flow characteristics of high-wax crude oil with temperature changes

**Figure 11:**
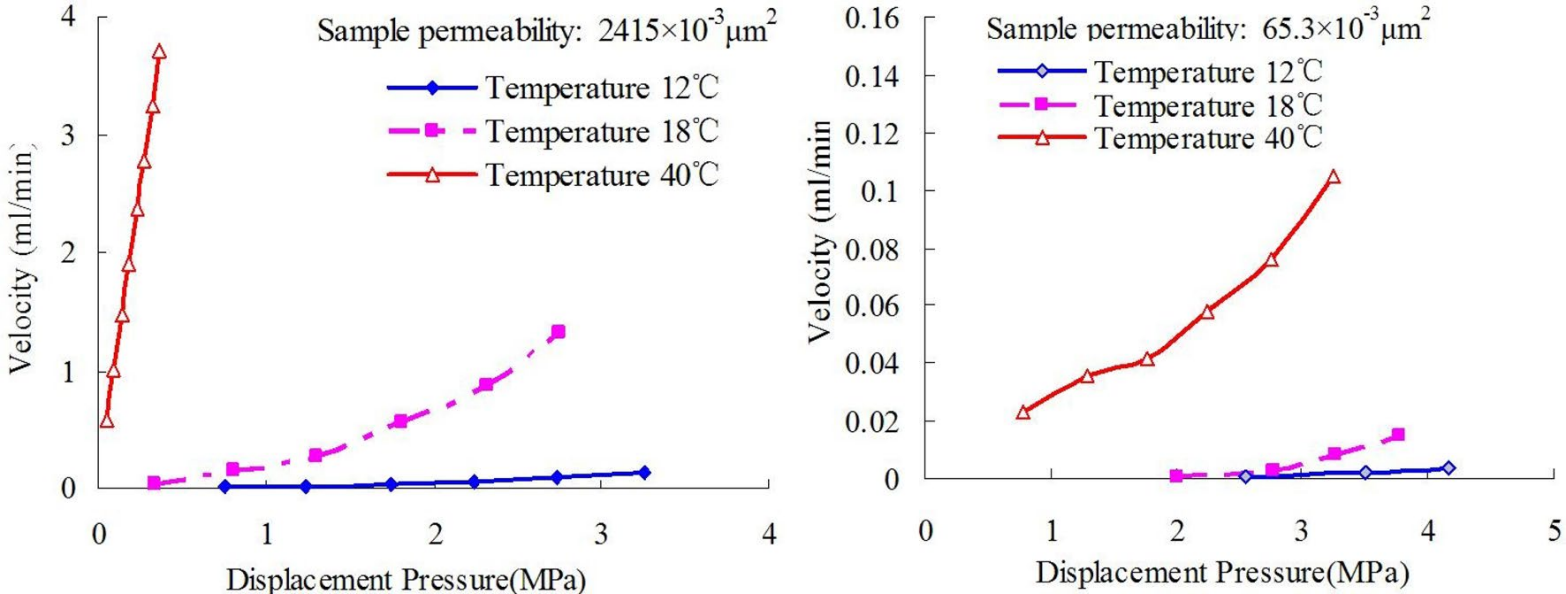
Displacement pressures changes in velocity at different temperatures.

For high-wax Changchunling Oilfield, a seepage flow experiment is conducted at varying temperatures by using the same set of cores. Figure 11 shows the contrast of core samples with different permeability with regard to displacement pressure and velocity at varying temperatures. The experimental result reveals that the displacement effect is enhanced as temperature increases gradually.

Changchunling Oilfield has adopted elastic displacement mining technology at the early period of exploitation. In addition, the threshold pressure gradient of high waxy crude is 6–10 times that of light oil, which indicates that wax deposition raises the threshold pressure gradient and makes the flow more difficult. Furthermore, wax deposition is more serious in low-pressure gradient conditions, resulting in high threshold pressure gradient.

### Quantitative analysis of wax precipitation damage to the reservoir

1. Influencing factors and changing characteristics of formation temperature.
  With the development of mining and the influence of water injection, the formation temperature gradually decreases, that is, within the short distance from the wellbore, the temperature changes greatly, and the change curve of temperature with depth far deviates from the geothermal static temperature curve^20^. According to the statistics of the production data of Changchunling oilfield, it is also found that the area where the reservoir wax precipitation is mainly within 15 m from the wellbore, which indicates that the temperature near the wellbore is relatively low. The average annual surface temperature of Changchunling oilfield is about 5 °C, and the top buried depth of the oil layer is 200–350 m. According to the analytical method in the literature^21^, it is found that the formation temperature near the well section is about 8 °C within the oil depth range of the study area, which is far lower than the wax freezing point temperature of 15.8 °C in this area. The above is the root cause of serious wax precipitation in this area.
2. Quantitative analysis of wax deposition in different wells.
  By analyzing the relationship curve between the volume fraction of wax precipitation and temperature in Fig. 6 in the text, it can be concluded that when the oil layer temperature of four wells C110, C107-3, C109 and C107-1-6 is 8 °C, the volume fraction of wax deposition is 4.47%, 6.67%, 4.9% and 7.4%.
3. Influence of wax deposition on porosity and permeability.
  How takes care of the precipitation of wax affect the porosity and permeability of the formation? The following is a quantitative analysis of the relationship between porosity, permeability and specific surface.

**Figure 12:**
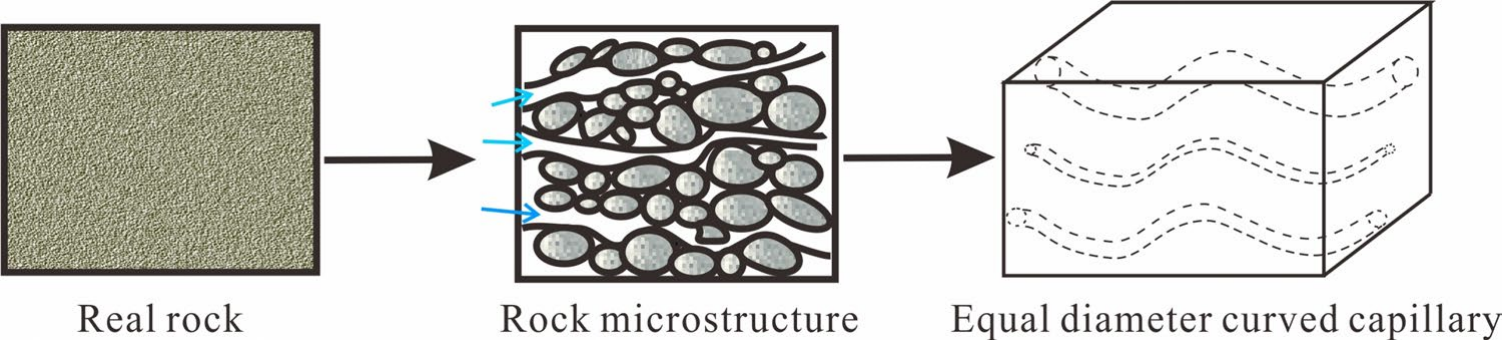
Simplified model of real reservoir rock geology.

The wax is deposited in the small pores of the formation. For the convenience of research, we take a small block of the reservoir rock to study and simplify the actual reservoir rock into a series of capillary bundles. As shown in Fig. 12.

Under the action of a certain stress state, the cross-sectional area of the rock is A and the length is L; the number of capillaries per unit area of the rock is n, the curvature of each capillary is τ, and r is the assumed capillary radius. According to the mercury injection data, the actual porous medium is equivalent to a capillary bundle model composed of a cluster of capillaries with different pore radius^22^. The high-pressure mercury intrusion experiment has been carried out on the core in the study area. According to the capillary pressure curve and the frequency distribution histogram of the pore throat radius, the average capillary radius r can be obtained.

In the case of the same external dimensions, the same fluid properties and the same acting pressure difference, the permeability k can be obtained from the Poiseuille equation and Darcy's law as follow^23^:

12 $$k=n\frac{\pi {r}^{4}}{8\tau }$$

Kozeny^24^ discussed the relationship between permeability and porosity, specific surface, etc. The porosity of the capillary rock model in Fig. 12 can be expressed as:

13 $$\Phi = \frac{{{\text{Pore}}\;{\text{volume}}}}{{{\text{Total}}\;{\text{volume}}\;{\text{of}}\;{\text{rock}}}} = \frac{{An\pi r^{2} L\tau }}{AL} = n\pi r^{2} \tau$$

Specific surface area is based on pore volume of rock:

14 $$S_{\Phi } = \frac{{{\text{Total}}\;{\text{surface}}\;{\text{area}}\;{\text{of}}\;{\text{rock}}\;{\text{particles}}}}{{{\text{Total}}\;{\text{volume}}\;{\text{of}}\;{\text{rock}}\;{\text{pore}}}} = \frac{{nA\left( {2\pi r} \right)L\tau }}{{nA\pi r^{2} L\tau }} = \frac{2}{\pi }$$

From Eqs. (12), (13) and (14), it can be obtained that:

15 $${S}_{\Phi }=\frac{1}{\tau }\sqrt{\frac{\Phi }{2k}}$$

16 $$k=\frac{\Phi {r}^{2}}{8{\tau }^{2}}$$

When wax deposition does not occur, the pore volume is V_Φ_. When wax deposition occurs, the wax adheres to the surface of the rock pores in the form of thin film, and the deposited volume is V_ω_. Assuming that the permeability and porosity of the formation become Φ′ and k′, and the capillary radius becomes r′, the specific surface area after wax precipitation is:

17 $$S_{\Phi } = \frac{{{\text{Total}}\;{\text{surface}}\;{\text{area}}\;{\text{of}}\;{\text{rock}}\;{\text{particles}}}}{{{\text{Total}}\;{\text{volume}}\;{\text{of}}\;{\text{rock}}\;{\text{pore}}}} = \frac{{nA\left( {2\pi r^{\prime}} \right)L\tau }}{{V_{\Phi } - V_{\omega } }}$$

Combined with Eqs. (15) and (17), the capillary radius r′ after wax deposition can be obtained:

18 $${r}^{\mathrm{^{\prime}}}=\frac{({V}_{\Phi }-{V}_{\omega })}{{\tau }^{2}(2\pi nAL)}\sqrt{\frac{\Phi }{2k}}=\frac{(\Phi V-{V}_{\omega })}{{\tau }^{2}(2\pi nV)}\sqrt{\frac{\Phi }{2k}}$$

Thus, the formation porosity Φ′ after wax deposition can be obtained:

19 $${\Phi }^{\mathrm{^{\prime}}}=n\pi {r\mathrm{^{\prime}}}^{2}\tau =n\pi \frac{{(\Phi V-{V}_{\omega })}^{2}}{{\tau }^{4}(4{n}^{2}{\pi }^{2}{V}^{2})}\frac{\Phi }{2k}\tau =\frac{{(\Phi V-{V}_{\omega })}^{2}}{{\tau }^{3}(n\pi {V}^{2})}\frac{\Phi }{8k}$$

By substituting Eqs. (13) and (16) into Eq. (19), the porosity after wax evolution can be changed into:

20 $${\Phi }^{\mathrm{^{\prime}}}=\frac{{(\Phi V-{V}_{\omega })}^{2}}{8{\tau }^{2}({V}^{2})}\frac{8{\tau }^{2}}{\Phi }=\frac{{(\Phi V-{V}_{\omega })}^{2}}{\Phi {V}^{2}}$$

From Eq. (15), the permeability k′ after wax evolution can be calculated as:

21 $${k}^{\mathrm{^{\prime}}}=\frac{\Phi \mathrm{^{\prime}}}{2{\tau }^{2}{S}_{\Phi }^{2}}$$

According to Eqs. (16) and (20), the permeability ratio before and after wax precipitation is:

22 $$\frac{k}{{k^{\prime}}} = \frac{{\Phi r^{2} }}{{\Phi^{\prime} r^{\prime 2} }}$$

23 $$\frac{{k^{\prime}}}{k} = \frac{{\Phi^{\prime}r^{\prime 2} }}{{\Phi r^{2} }} = \frac{{(\Phi {\text{V}} - {\text{V}}_{\omega } )^{2} }}{{\Phi^{2} V^{2} }} \cdot \frac{{r^{\prime 2} }}{{r^{2} }}$$

Let $m = \frac{{k^{\prime}}}{k}$, assuming that the volume percentage of wax deposition in total pores is b, then Eqs. (20) and (23) become:

24 $${\Phi }^{\mathrm{^{\prime}}}=\frac{{(\Phi -\mathrm{b})}^{2}}{\Phi }$$

25 $$m=\frac{(\Phi - \text{b} {)}^{2}}{{\Phi }^{2}}\cdot (1-b)$$

**Figure 13:**
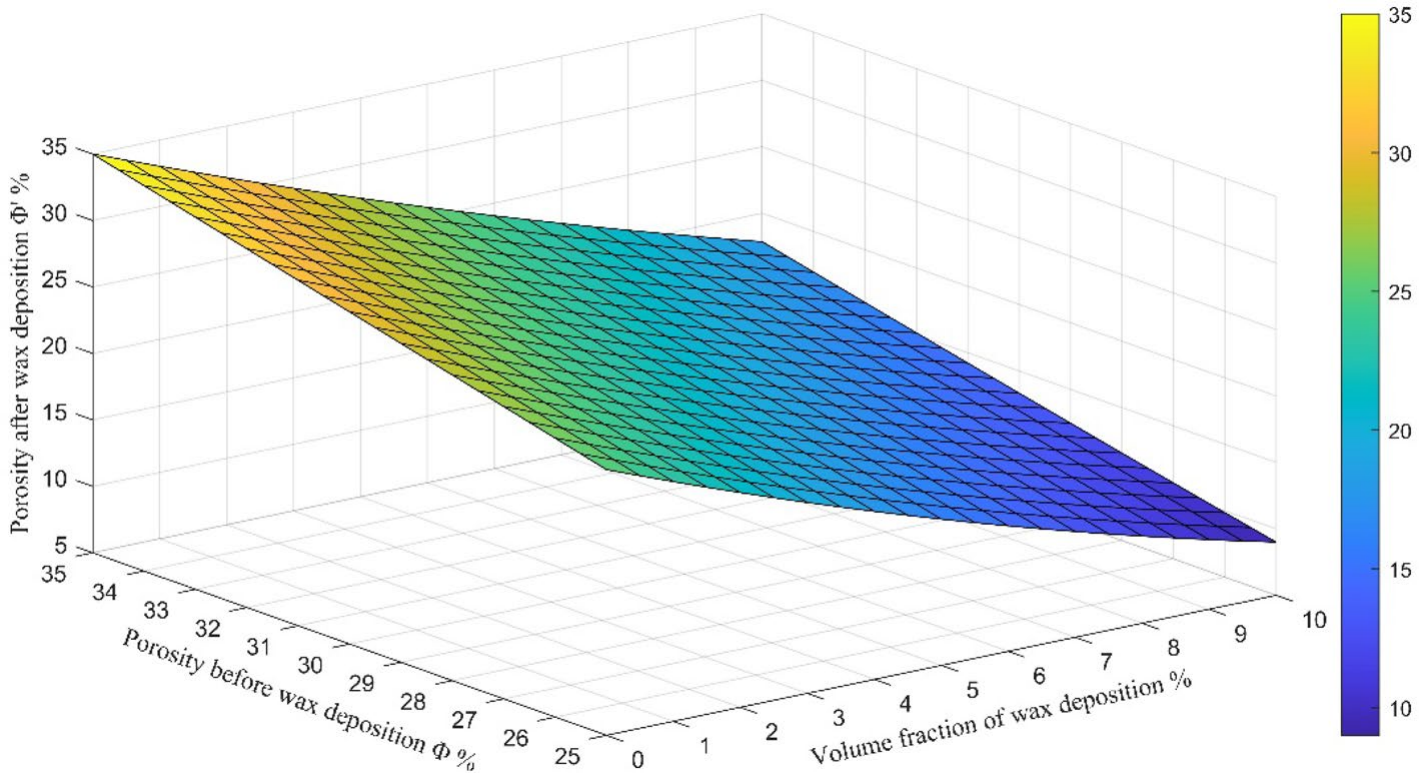
Relation curve of the porosity after wax deposition and wax precipitation volume fraction and the original porosity.

According to Eq. (24), the curve of the porosity after wax deposition and wax precipitation volume fraction and the original porosity are obtained, as showed in Fig. 13.

**Figure 14:**
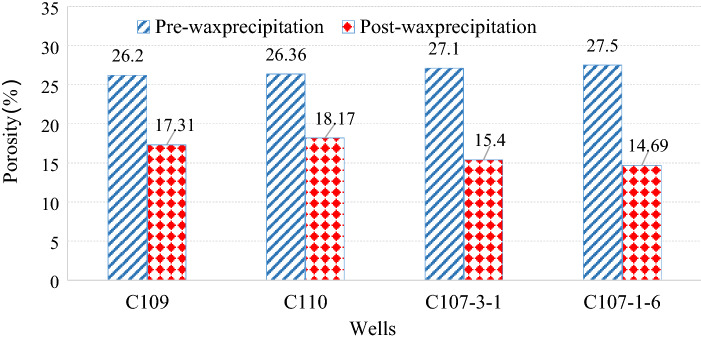
The porosity changes of four wells before and after wax deposition.

It can be seen from Fig. 13 that the rock porosity after wax precipitation decreases with the increase of the volume fraction of wax precipitation and increases with the increase of the original reservoir porosity. As mentioned above, according to the volume fraction of wax deposition in four wells C109, C110, C107-3-1 and C107-1-6 from Fig. 6: 4.9%, 4.47%, 6.67% and 7.4%, the porosity after the paraffin deposits decreased from the original porosity of 26.2%, 26.36%, 27.1% and 27.5% to 17.31%, 18.17%, 15.4% and 14.69%, as showed in Fig. 14. The damage to the reservoir caused by wax deposition is very serious.

**Figure 15:**
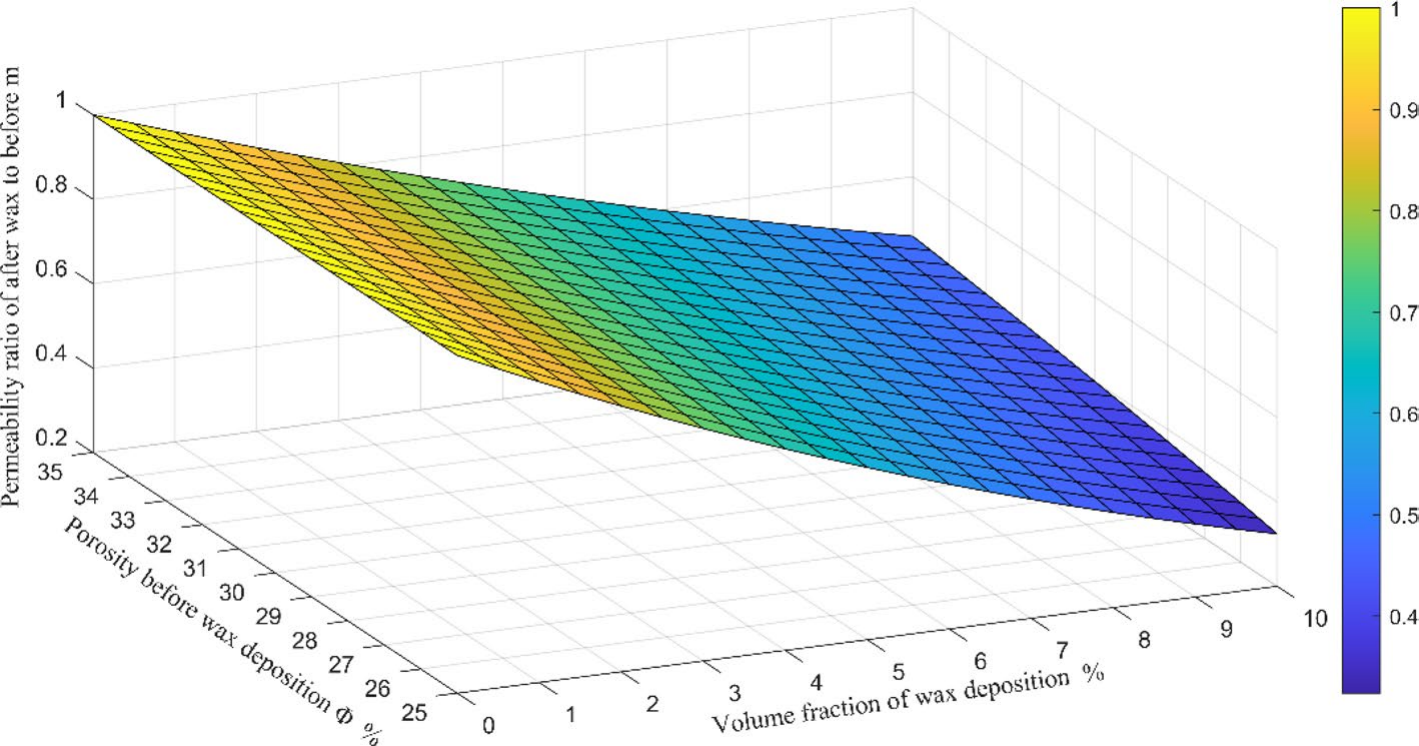
Relation curve of permeability ratio of after wax to before, volume fraction of wax deposition and porosity before wax deposition.

From Eq. (25), the curve of permeability ratio of after waxing to before m, volume fraction of wax deposition and porosity before wax deposition is showed in Fig. 15.

**Figure 16:**
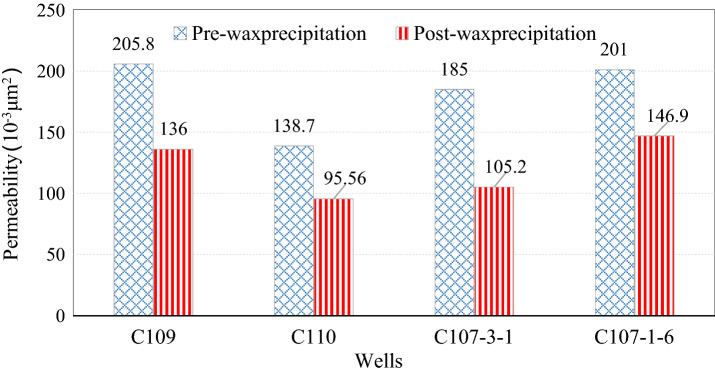
The permeability changes of four wells before and after wax deposition.

It can be seen from Fig. 15 that the permeability ratio (after and before wax precipitation) is less than 1, and decreases with the increase of wax precipitation volume fraction, and increases with the increase of the original reservoir porosity. As mentioned above, according to the four wells C109, C110, C107-3-1 and C107-1-6, the original porosity is 26.2%, 26.36%, 27.1% and 27.5%; the permeability changes from 205.8, 138.7, 185, and 201 (10^–3^ μm^2^) before wax precipitation to 136, 95.56, 105.2, and 146.9 (10^–3^ μm^2^) after wax deposition. Permeability changes of the four wells are shown in Fig. 16.

### Relative permeability–temperature curve of high-wax crude oil

**Figure 17:**
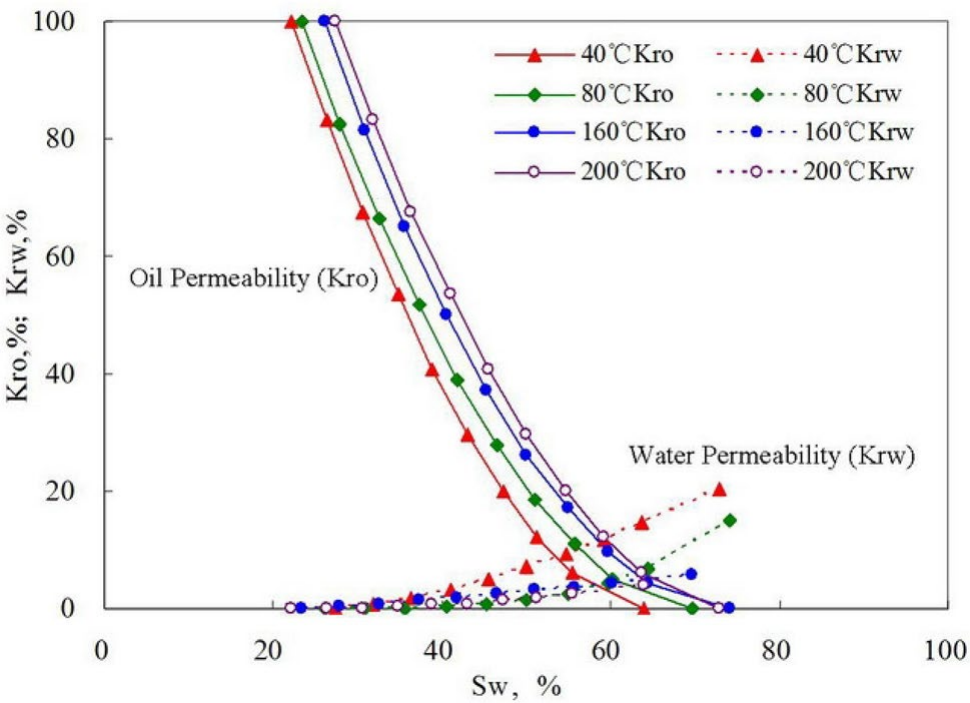
Relative permeability–temperature curves of oil and water.

Characteristics of oil–water seepage flow fundamentally affect the water drive characteristics in a reservoir^25,26^. The study of oil–water seepage flow characteristics is of theoretical and practical significance in understanding the essence of the water drive curve. For high-wax oil reservoirs, oilfield development is directly affected by the variation in oil–water relative permeability. The rheological characteristics of crude oil are very poor in Changchunling Oilfield because of wax deposition^27–29^. As to two-phase oil–water flow, the relative permeability of the oil phase is not only affected by the porous medium but also closely related to crude oil properties^30^. According to the results of numerous indoor displacement experiments, the two-phase oil–water relative permeability curve at varying temperatures is given in Fig. 17. In water flooding experiment, the relative permeability of the oil phase slowly decreased with the gradual increase in water saturation. In sum, crude oil exhibits a certain seepage property even in the condition of low oil saturation. When the experimental temperature decreases from 200 to 40 °C, the oil phase permeability sharply decreased as oil saturation is reduced. When the formation temperature drops lower than the pour point of wax deposition, the oil phase permeability drops rapidly. As crude oil viscosity increase and the flowing space are narrowed, oil phase permeability declines substantially, while the relative permeability of water phase slowly decreases with the gradual increase of the temperature from 40 to 200 °C. The mechanism is as follows:

High temperatures, lipophilic polar substances desorb from the rock surface, and large amounts of water adsorbs on the rock surface instead. The original oil channel turns into a water channel. Therefore, the temperature of the injected water must be higher than that of wax deposition in water flooding exploitation.

**Figure 18:**
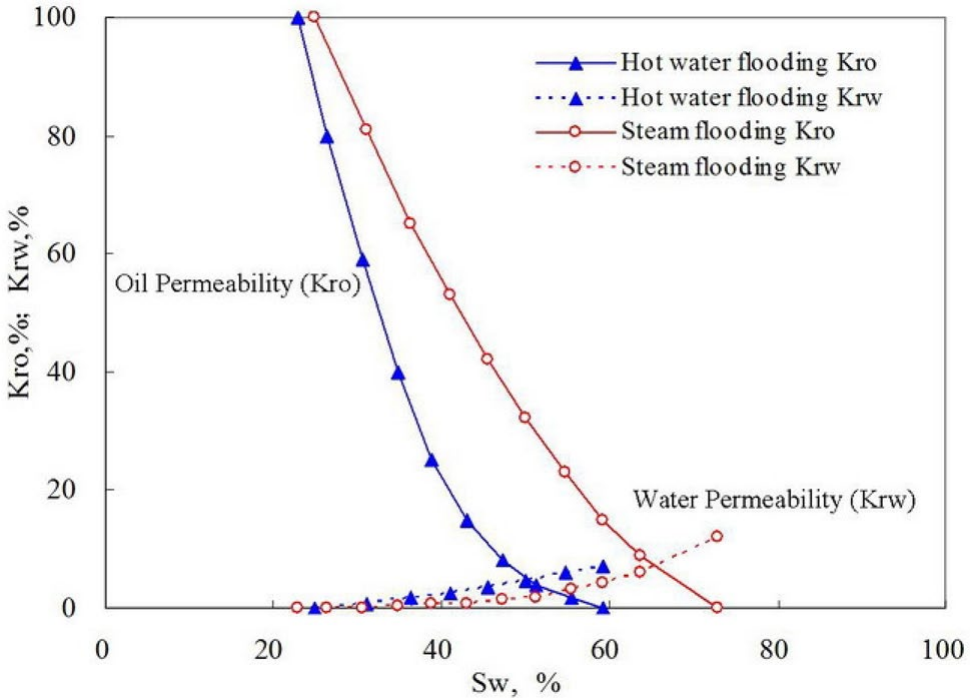
Curves of relative permeability of water flooding and steam flooding.

Figure 18 shows the comparison of relative permeability of water flooding and stream flooding. Generally, the relative permeability of steam flooding is higher than that of hot water flooding. The permeability value corresponding to isotonic point is higher, the two-phase flow area is larger. From hot water flooding to steam flooding, the wettability of a reservoir tends to be more hydrophilic, which induces a better oil seepage. This may be significant in enhancing the ultimate recovery. Therefore, hot water flooding or steam flooding can improve the development efficiency of high-wax reservoir.

## Conclusion

1. Wax deposition is an important factor leading to oil effective permeability and well productivity drop.
2. As soon as the formation temperature drops below the wax appearance point, wax deposition begins to occur. The effective permeability of oil decreases with the drop in temperature and this damage is irreversible.
3. When the temperature is between pour point and wax appearance point, wax crystal appears in waxy crude, and then filtration or adhesion happens in the pore. Eventually, the porosity falls gradually.
4. Steam or warm water injection is an effective method to keep high temperature and prevent wax deposition. The experimental results show that the relative permeability of steam flooding is higher than that of hot water flooding. The permeability value corresponding to isotonic point is higher, the two-phase flow area is larger. From hot water flooding to steam flooding, the wettability of a reservoir tends to be more hydrophilic, which induces a better oil seepage.
